# The genome sequence of the Brindled Flat-body, *Agonopterix arenella* (Denis & Schiffermüller, 1775)

**DOI:** 10.12688/wellcomeopenres.19252.1

**Published:** 2023-05-12

**Authors:** Douglas Boyes, Peter W.H. Holland, University of Oxford and Wytham Woods Genome Acquisition Lab, Darwin Tree of Life Barcoding collective, Wellcome Sanger Institute Tree of Life programme, Wellcome Sanger Institute Scientific Operations: DNA Pipelines collective, Tree of Life Core Informatics collective, Darwin Tree of Life Consortium

**Affiliations:** 1 UK Centre for Ecology & Hydrology, Wallingford, England, UK; 2 University of Oxford, Oxford, England, UK

**Keywords:** Agonopterix arenella, Brindled Flat-body, genome sequence, chromosomal, Lepidoptera

## Abstract

We present a genome assembly from an individual male *Agonopterix arenella* (the Brindled Flat-body; Arthropoda; Insecta; Lepidoptera; Depressariidae). The genome sequence is 545.8 megabases in span. Most of the assembly is scaffolded into 30 chromosomal pseudomolecules, including the assembled Z sex chromosome. The mitochondrial genome has also been assembled and is 15.3 kilobases in length.

## Species taxonomy

Eukaryota; Metazoa; Ecdysozoa; Arthropoda; Hexapoda; Insecta; Pterygota; Neoptera; Endopterygota; Lepidoptera; Glossata; Ditrysia; Gelechioidea; Depressariidae; Depressariinae; *Agonopterix*; *Agonopterix arenella* (Denis & Schiffermüller, 1775) (NCBI:txid1594222).

## Background

Moths in the genus *Agonopterix* have a flattened oval shape when at rest, formed by overlapping their rounded wings directly above a compressed abdomen. Many *Agonopterix* species hibernate as adults, and the flat shape may facilitate hiding in bark crevices or leaf litter ( Harper *et al.*, 2002). Some species in the genus can be difficult to distinguish as adults; detailed descriptions and useful taxonomic keys, based on species found in the Netherlands, have been published ( Huisman, 2012).

*Agonopterix arenella,* sometimes called the Brindled Flat-body, is a common moth found widely across northern Europe and Scandinavia, including most of Britain and Ireland ( GBIF Secretariat, 2022; Harper *et al.,* 2002; Sterling & Parsons, 2018). The adult moth has buff-coloured forewings marked with a brown ‘smudged’ blotch and several sharper brown-black dots forming a paw print impression. *A. arenella* is attracted to light and adults can be recorded throughout the year, with numbers peaking sharply in autumn and spring before and after hibernation ( Asher *et al.*, 2013; Huisman, 2012; NBN Atlas, 2021). Eggs are laid in spring on the leaves of the food plant, usually thistles *Carduus* and *Cirsium* spp., knapweeds *Centaurea* spp., burdocks *Arctium* spp. or saw-wort *Serratula tinctoria*. The larvae initially mine inside the leaf blade before spinning a feeding web on the underside of leaves ( Harper *et al.*, 2002). The pupal stage is brief before the adults emerge in autumn.

An assembled genome sequence for *A. arenella* will contribute to the growing set of genomic resources for understanding lepidopteran biology.

### Genome sequence report

**Figure 1.**
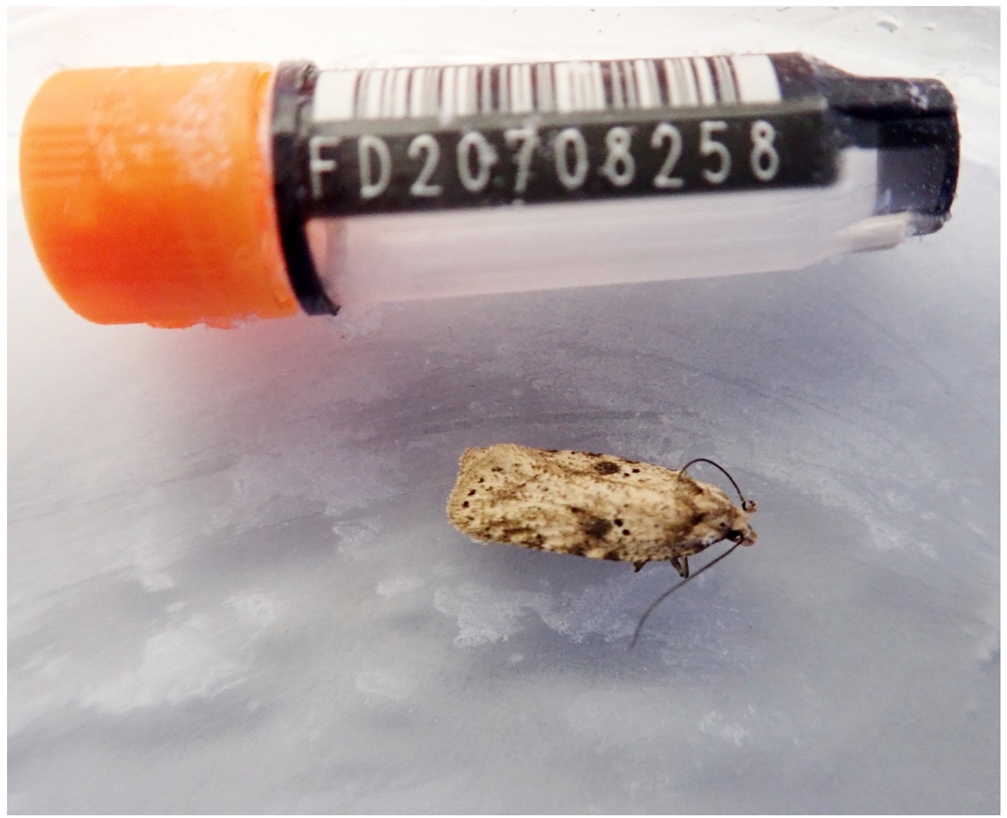
Photograph of the *Agonopterix arenella* (ilAgoAren1) specimen used for genome sequencing.

The genome was sequenced from one male *A. arenella* ( Figure 1) collected from Wytham Woods, Oxfordshire, UK (latitude 51.77, longitude –1.34). A total of 42-fold coverage in Pacific Biosciences single-molecule HiFi long reads and 79-fold coverage in 10X Genomics read clouds were generated. Primary assembly contigs were scaffolded with chromosome conformation Hi-C data. Manual assembly curation corrected seven missing joins or mis-joins and removed three haplotypic duplications, reducing the assembly length by 1.93% and the scaffold number by 15.79%, and increasing the scaffold N50 by –2.06%.

**Figure 2.**
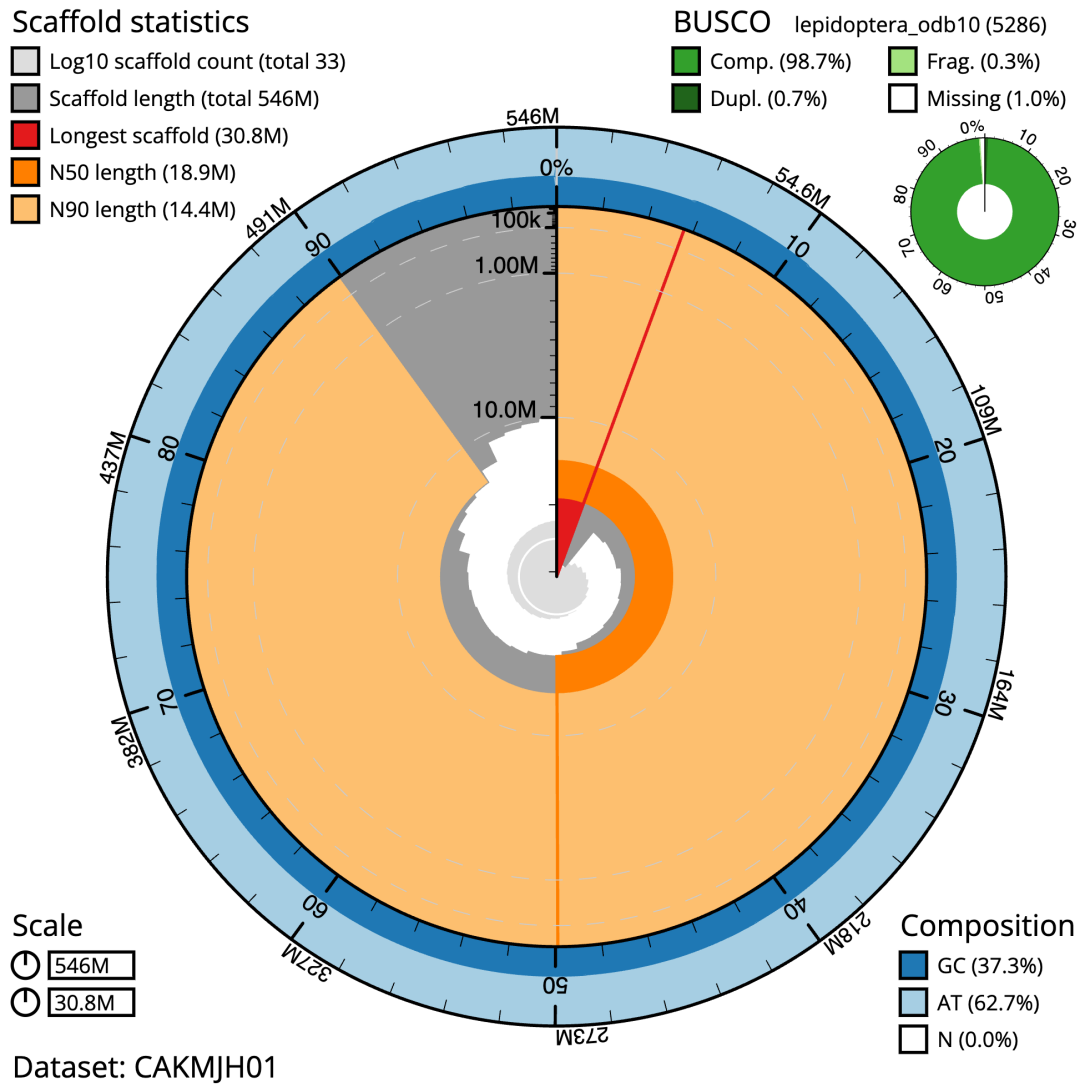
Genome assembly of *Agonopterix arenella*, ilAgoAren1.1: metrics. The BlobToolKit Snailplot shows N50 metrics and BUSCO gene completeness. The main plot is divided into 1,000 size-ordered bins around the circumference with each bin representing 0.1% of the 545,825,857 bp assembly. The distribution of scaffold lengths is shown in dark grey with the plot radius scaled to the longest scaffold present in the assembly (30,785,055 bp, shown in red). Orange and pale-orange arcs show the N50 and N90 scaffold lengths (18,923,089 and 14,436,485 bp), respectively. The pale grey spiral shows the cumulative scaffold count on a log scale with white scale lines showing successive orders of magnitude. The blue and pale-blue area around the outside of the plot shows the distribution of GC, AT and N percentages in the same bins as the inner plot. A summary of complete, fragmented, duplicated and missing BUSCO genes in the lepidoptera_odb10 set is shown in the top right. An interactive version of this figure is available at https://blobtoolkit.genomehubs.org/view/ilAgoAren1.1/dataset/CAKMJH01/snail.

The final assembly has a total length of 545.8 Mb in 32 sequence scaffolds with a scaffold N50 of 18.9 Mb ( Table 1). Most (99.98%) of the assembly sequence was assigned to 30 chromosomal-level scaffolds, representing 29 autosomes, and the Z sex chromosome. Chromosome-scale scaffolds confirmed by the Hi-C data are named in order of size ( Figure 2– Figure 5; Table 2). While not fully phased, the assembly deposited is of one haplotype. Contigs corresponding to the second haplotype have also been deposited. The estimated Quality Value (QV) of the final assembly is 60.2 with *k*-mer based completeness of 100%, and the assembly has a BUSCO v5.3.2 ( Manni *et al.*, 2021) completeness of 98.7% (single 98.0%, duplicated 0.7%) using the lepidoptera_odb10 reference set ( *n* = 5,286).

**Table 1.** Genome data for *Agonopterix arenella*, ilAgoAren1.1.

Project accession data		
Assembly identifier	ilAgoAren1.1	
Species	*Agonopterix arenella*	
Specimen	ilAgoAren1	
NCBI taxonomy ID	1594222	
BioProject	PRJEB47542	
BioSample ID	SAMEA8603192	
Isolate information	ilAgoAren1	
Assembly metrics *		*Benchmark*
Consensus quality (QV)	60.2	*≥ 50*
*k*-mer completeness	100%	*≥ 95%*
BUSCO **	C:98.7%[S:98.0%,D:0.7%], F:0.3%,M:1.0%,n:5,286	*C ≥ 95%*
Percentage of assembly mapped to chromosomes	99.98%	*≥ 95%*
Sex chromosomes	Z chromosome assembled	*localised homologous pairs*
Organelles	Mitochondrial genome assembled	*complete single alleles*
Raw data accessions		
PacificBiosciences SEQUEL II	ERR6909091, ERR6939288	
10X Genomics Illumina	ERR6787432–ERR6787435	
Hi-C Illumina	ERR6787431	
Genome assembly		
Assembly accession	GCA_927399405.1	
*Accession of alternate haplotype*	GCA_927400025.1	
Span (Mb)	545.8	
Number of contigs	39	
Contig N50 length (Mb)	18.9	
Number of scaffolds	32	
Scaffold N50 length (Mb)	18.9	
Longest scaffold (Mb)	30.8	

* Assembly metric benchmarks are adapted from column VGP-2020 of “Table 1: Proposed standards and metrics for defining genome assembly quality” from ( Rhie *et al.*, 2021). ** BUSCO scores based on the lepidoptera_odb10 BUSCO set using v5.3.2. C = complete [S = single copy, D = duplicated], F = fragmented, M = missing, n = number of orthologues in comparison. A full set of BUSCO scores is available at https://blobtoolkit.genomehubs.org/view/ilAgoAren1.1/dataset/CAKMJH01/busco.

**Figure 3.**
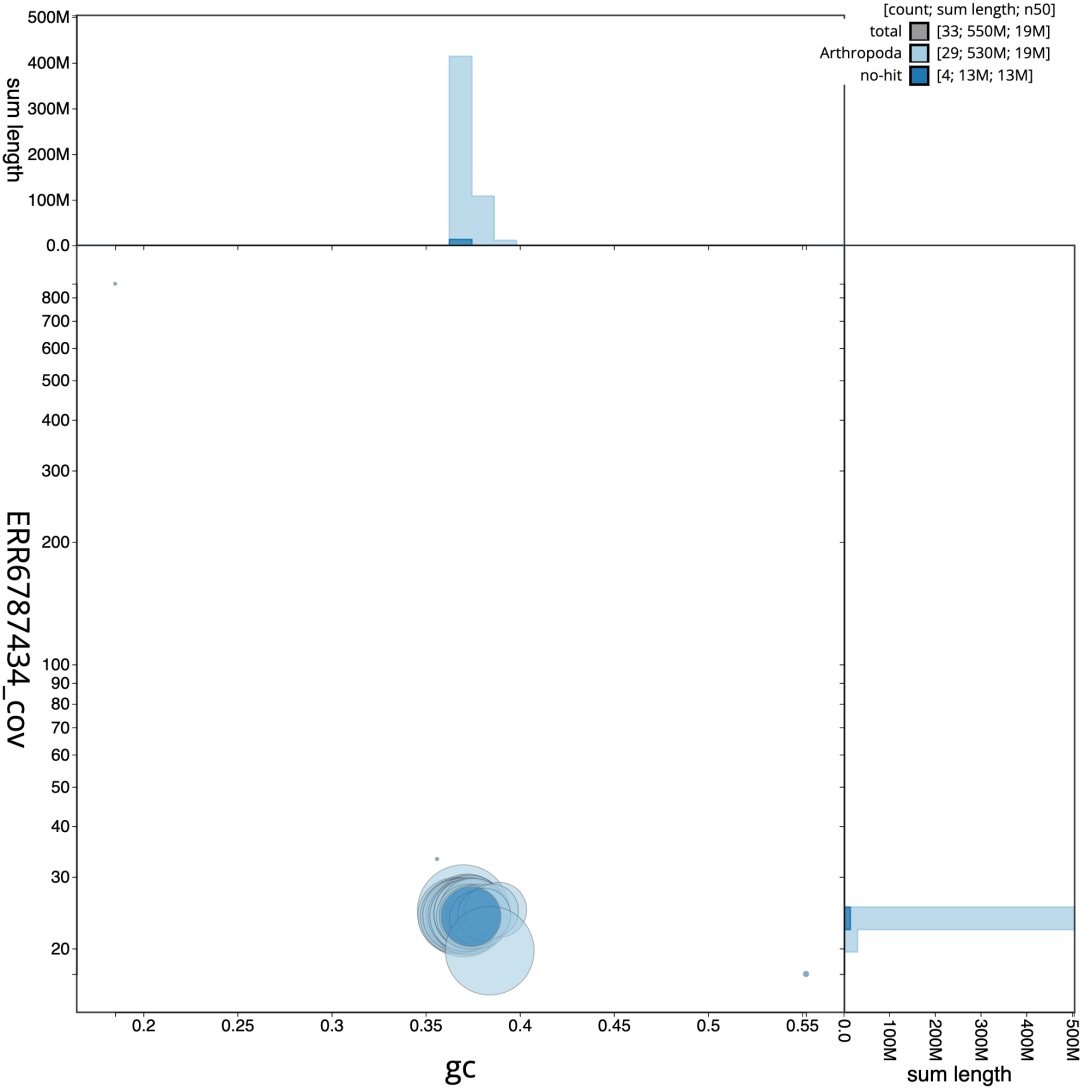
Genome assembly of *Agonopterix arenella*, ilAgoAren1.1: GC coverage. BlobToolKit GC-coverage plot. Scaffolds are coloured by phylum. Circles are sized in proportion to scaffold length. Histograms show the distribution of scaffold length sum along each axis. An interactive version of this figure is available at https://blobtoolkit.genomehubs.org/view/ilAgoAren1.1/dataset/CAKMJH01/blob.

**Figure 4.**
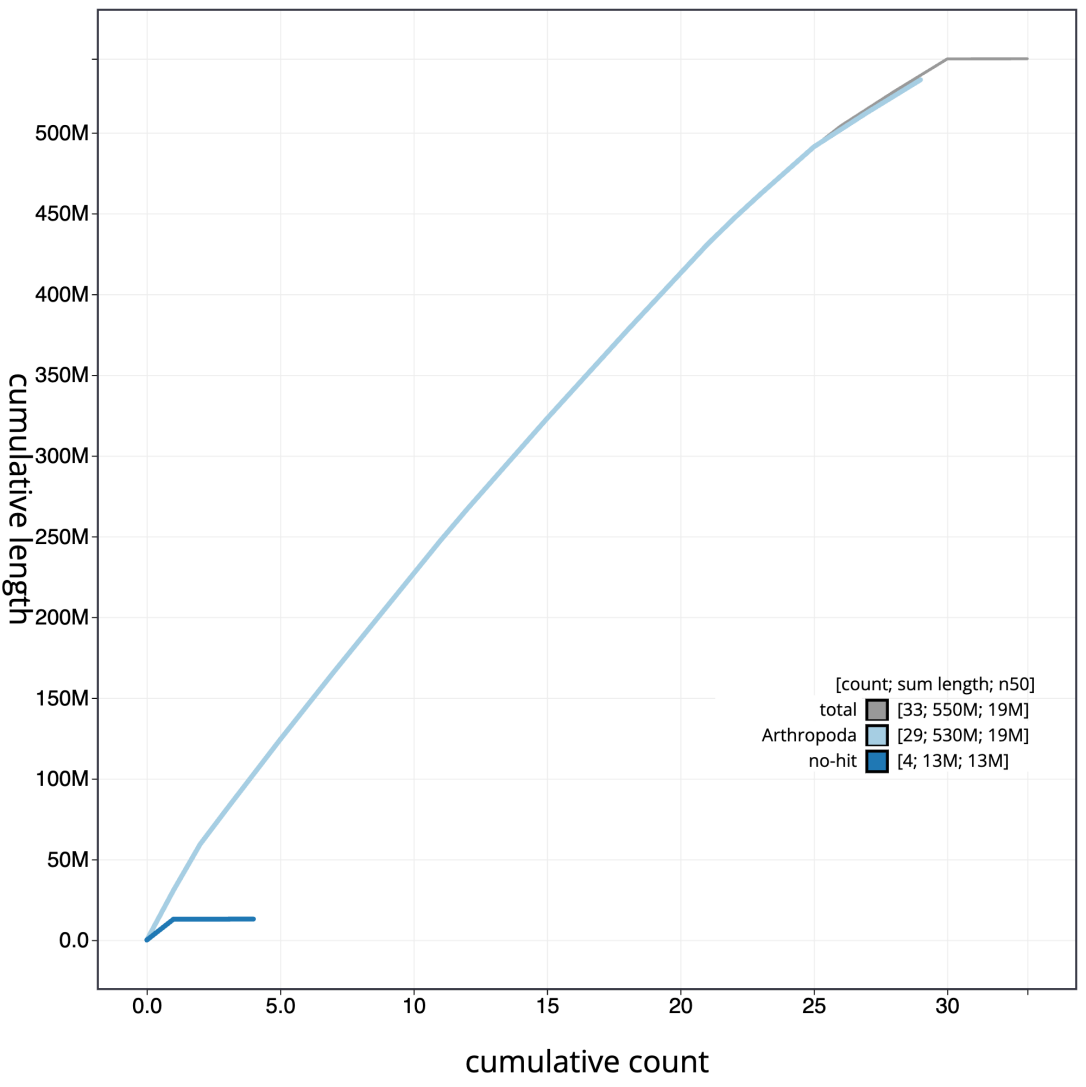
Genome assembly of *Agonopterix arenella*, ilAgoAren1.1: cumulative sequence. BlobToolKit cumulative sequence plot. The grey line shows cumulative length for all scaffolds. Coloured lines show cumulative lengths of scaffolds assigned to each phylum using the buscogenes taxrule. An interactive version of this figure is available at https://blobtoolkit.genomehubs.org/view/ilAgoAren1.1/dataset/CAKMJH01/cumulative.

**Figure 5.**
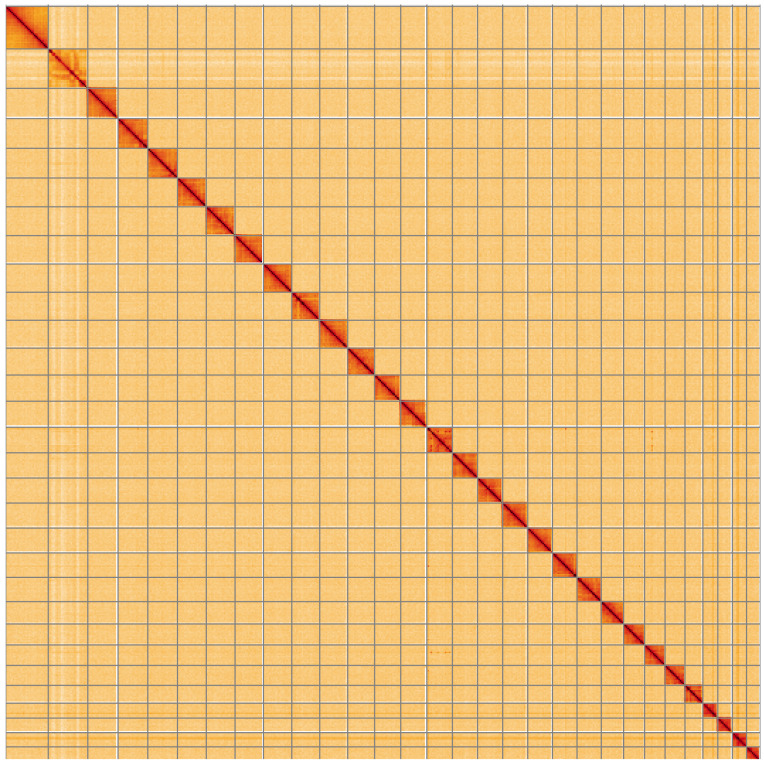
Genome assembly of *Agonopterix arenella*, ilAgoAren1.1: Hi-C contact map. Hi-C contact map of the ilAgoAren1.1 assembly, visualised using HiGlass. Chromosomes are shown in order of size from left to right and top to bottom. An interactive version of this figure may be viewed at https://genome-note-higlass.tol.sanger.ac.uk/l/?d=eBh637UMSs-8lwIy7MFttg.

**Table 2.** Chromosomal pseudomolecules in the genome assembly of *Agonopterix arenella*, ilAgoAren1.

INSDC accession	Chromosome	Size (Mb)	GC%
OV656689.1	1	30.79	37
OV656691.1	2	21.86	37.1
OV656692.1	3	21.53	37.2
OV656693.1	4	21.45	37.3
OV656694.1	5	20.87	37.3
OV656695.1	6	20.8	36.6
OV656696.1	7	20.54	36.8
OV656697.1	8	20.46	37
OV656698.1	9	20.26	36.7
OV656699.1	10	20.19	37
OV656700.1	11	19.44	36.8
OV656701.1	12	18.92	37.2
OV656702.1	13	18.87	37.2
OV656703.1	14	18.46	37.2
OV656704.1	15	18.25	37.1
OV656705.1	16	18.11	37
OV656706.1	17	18.11	37.1
OV656707.1	18	17.94	37.3
OV656708.1	19	17.72	37.3
OV656709.1	20	17.54	37.6
OV656710.1	21	16.16	37.5
OV656711.1	22	15.18	37.3
OV656712.1	23	14.79	37.8
OV656713.1	24	14.44	37.4
OV656714.1	25	12.92	37.4
OV656715.1	26	10.77	38.9
OV656716.1	27	10.53	37.7
OV656717.1	28	10.27	38.5
OV656718.1	29	9.98	38.1
OV656690.1	Z	28.58	38.4
OV656719.1	MT	0.02	18.8
-	unplaced	0.09	50.6

## Methods

### Sample acquisition and nucleic acid extraction

A male *A. arenella* specimen (ilAgoAren1) was collected from Wytham Woods, Oxfordshire (biological vice-county Berkshire), UK (latitude 51.77, longitude –1.34) on 8 September 2020. The specimen was taken from woodland habitat by Douglas Boyes (University of Oxford) using a light trap. The specimen was identified by the collector and snap-frozen on dry ice.

DNA was extracted at the Tree of Life laboratory, Wellcome Sanger Institute (WSI). The ilAgoAren1 sample was weighed and dissected on dry ice with tissue set aside for Hi-C sequencing. Whole organism tissue was disrupted using a Nippi Powermasher fitted with a BioMasher pestle. High molecular weight (HMW) DNA was extracted using the Qiagen MagAttract HMW DNA extraction kit. Low molecular weight DNA was removed from a 20 ng aliquot of extracted DNA using the 0.8X AMpure XP purification kit prior to 10X Chromium sequencing; a minimum of 50 ng DNA was submitted for 10X sequencing. HMW DNA was sheared into an average fragment size of 12–20 kb in a Megaruptor 3 system with speed setting 30. Sheared DNA was purified by solid-phase reversible immobilisation using AMPure PB beads with a 1.8X ratio of beads to sample to remove the shorter fragments and concentrate the DNA sample. The concentration of the sheared and purified DNA was assessed using a Nanodrop spectrophotometer and Qubit Fluorometer and Qubit dsDNA High Sensitivity Assay kit. Fragment size distribution was evaluated by running the sample on the FemtoPulse system.

### Sequencing

Pacific Biosciences HiFi circular consensus and 10X Genomics read cloud DNA sequencing libraries were constructed according to the manufacturers’ instructions. DNA sequencing was performed by the Scientific Operations core at the WSI on the Pacific Biosciences SEQUEL II (HiFi) and Illumina NovaSeq 6000 (10X) instruments. Hi-C data were also generated from tissue of ilAgoAren1 using the Arima v2 kit and sequenced on the Illumina NovaSeq 6000 instrument.

### Genome assembly

Assembly was carried out with Hifiasm ( Cheng *et al.*, 2021) and haplotypic duplication was identified and removed with purge_dups ( Guan *et al.*, 2020). One round of polishing was performed by aligning 10X Genomics read data to the assembly with Long Ranger ALIGN, calling variants with FreeBayes ( Garrison & Marth, 2012). The assembly was then scaffolded with Hi-C data ( Rao *et al.*, 2014) using SALSA2 ( Ghurye *et al.*, 2019). The assembly was checked for contamination as described previously ( Howe *et al.*, 2021). Manual curation was performed using HiGlass ( Kerpedjiev *et al.*, 2018) and Pretext ( Harry, 2022). The mitochondrial genome was assembled using MitoHiFi ( Uliano-Silva *et al.*, 2022), which performed annotation using MitoFinder ( Allio *et al.*, 2020). To evaluate the assembly, MerquryFK was used to estimate consensus quality (QV) scores and *k*-mer completeness ( Rhie *et al.*, 2020). The genome was analysed, and BUSCO scores ( Manni *et al.*, 2021; Simão *et al.*, 2015) were generated within the BlobToolKit environment ( Challis *et al.*, 2020). Table 3 contains a list of software tool versions and sources.

**Table 3.** Software tools and versions used.

Software tool	Version	Source
BlobToolKit	4.0.7	https://github.com/blobtoolkit/blobtoolkit
BUSCO	5.3.2	https://gitlab.com/ezlab/busco
FreeBayes	1.3.1-17-gaa2ace8	https://github.com/freebayes/freebayes
Hifiasm	0.12	https://github.com/chhylp123/hifiasm
HiGlass	1.11.6	https://github.com/higlass/higlass
Long Ranger ALIGN	2.2.2	https://support.10xgenomics.com/genome-exome/ software/pipelines/latest/advanced/other-pipelines
Merqury	MerquryFK	https://github.com/thegenemyers/MERQURY.FK
MitoHiFi	2	https://github.com/marcelauliano/MitoHiFi
PretextView	0.2	https://github.com/wtsi-hpag/PretextView
purge_dups	1.2.3	https://github.com/dfguan/purge_dups
SALSA	2.2	https://github.com/salsa-rs/salsa

### Ethics and compliance issues

The materials that have contributed to this genome note have been supplied by a Darwin Tree of Life Partner. The submission of materials by a Darwin Tree of Life Partner is subject to the Darwin Tree of Life Project Sampling Code of Practice. By agreeing with and signing up to the Sampling Code of Practice, the Darwin Tree of Life Partner agrees they will meet the legal and ethical requirements and standards set out within this document in respect of all samples acquired for, and supplied to, the Darwin Tree of Life Project. All efforts are undertaken to minimise the suffering of animals used for sequencing. Each transfer of samples is further undertaken according to a Research Collaboration Agreement or Material Transfer Agreement entered into by the Darwin Tree of Life Partner, Genome Research Limited (operating as the Wellcome Sanger Institute), and in some circumstances other Darwin Tree of Life collaborators.

## Data Availability

European Nucleotide Archive: *Agonopterix arenella* (brindled flat-body). Accession number PRJEB47542; https://identifiers.org/ena.embl/PRJEB47542. ( Wellcome Sanger Institute, 2022) The genome sequence is released openly for reuse. The *Agonopterix arenella* genome sequencing initiative is part of the Darwin Tree of Life (DToL) project. All raw sequence data and the assembly have been deposited in INSDC databases. The genome will be annotated using available RNA-Seq data and presented through the Ensembl pipeline at the European Bioinformatics Institute. Raw data and assembly accession identifiers are reported in Table 1.

## Author information

Members of the University of Oxford and Wytham Woods Genome Acquisition Lab are listed here: https://doi.org/10.5281/zenodo.4789928.

Members of the Darwin Tree of Life Barcoding collective are listed here: https://doi.org/10.5281/zenodo.4893703.

Members of the Wellcome Sanger Institute Tree of Life programme are listed here: https://doi.org/10.5281/zenodo.4783585.

Members of Wellcome Sanger Institute Scientific Operations: DNA Pipelines collective are listed here: https://doi.org/10.5281/zenodo.4790455.

Members of the Tree of Life Core Informatics collective are listed here: https://doi.org/10.5281/zenodo.5013541.

Members of the Darwin Tree of Life Consortium are listed here: https://doi.org/10.5281/zenodo.4783558.
