# Supplementary material for: The genome sequence of the Brindled Flat-body, Agonopterix arenella (Denis & Schiffermüller, 1775)
Source: Wellcome Open Res. 2023 May 12;8:214. [Version 1] doi: 10.12688/wellcomeopenres.19252.1 (PMC11387930; doi:10.12688/wellcomeopenres.19252.1)
Supplement: Peer review history

# Reviewer report: Reviewer response for version 1

**DOI:** 10.21956/wellcomeopenres.21335.r96022

**Published:** 2024-09-10

**Authors:** Luca Livraghi

**Affiliations:** 1 George Washington University, Washington, USA

The paper reports a very high quality assembly generated from an individual male *Agonopterix arenella* moth. Some basic biology of the moth is discussed, as well as the rationale for sequencing. The genome generated is of excellent quality, with almost complete chromosomal scaffolding, large N50 and very high BUSCO completeness scores. This includes 30 chromosomal level scaffolds, including the Z chromosome. The assembly has no signs of contaminants and a GC% content consistent with other high quality lepidopteran assemblies.

 This is another fantastic addition to the ever-growing repertoire of lepidopteran genomes assembled by DToL, and will not doubt be useful to the community for comparative genomics studies. 

 Some minor comments:

 1. The methods mention the sample was collected on 8 September 2020 and snap frozen on dry ice, but storage conditions post snap freeze are not detailed (any buffer? dry storage at -80? How long was the sample stored before DNA extraction?).

 2. The methods lack some detail on what tissue was used for which library preparation. It mentions "tissue set aside for Hi-C seqeuncing." The methods could benefit from detailing the origin of the tissue for each library prep, if information is available (e.g 10X library from full organism, HiFi from subset of DNA extraction, Hi-C from subset of tissue x). 

 3. There is no mention of genome annotation methods beyond the mitochondrial genome. The ENA entry mentions "transcroptomic data." Was this collected for genome annotation? If so from which tissues, and how was this used to annotate the genome? If the genome will be annotated at a later date it would be helpful to add a note. This is mentioned in the Data availability but not immediately clear from the text.  

Are sufficient details of methods and materials provided to allow replication by others?

Partly

Is the rationale for creating the dataset(s) clearly described?

Yes

Are the datasets clearly presented in a useable and accessible format?

Partly

Are the protocols appropriate and is the work technically sound?

Yes

Reviewer Expertise:

Evolutionary developmental biology.

I confirm that I have read this submission and believe that I have an appropriate level of expertise to confirm that it is of an acceptable scientific standard.

# Reviewer report: Reviewer response for version 1

**DOI:** 10.21956/wellcomeopenres.21335.r57466

**Published:** 2023-05-26

**Authors:** Michael Hiller

**Affiliations:** 1 LOEWE-Centre for Translational Biodiversity Genomics (TBG), Senckenberg Nature Research Society, Frankfurt Am Main, Germany

The data note presents a high quality genome of the moth *Agonopterix arenella. *The assembly is of exceptional quality, with high contig N50 values, chromosome-level scaffolds and a high base accuracy (QV60). This genome adds to genomic resources that will enable comparative lepidopteran studies.

 The manuscript is well written and the methods are clearly described. 

 I have no comments and hence recommend indexing.

Are sufficient details of methods and materials provided to allow replication by others?

Yes

Is the rationale for creating the dataset(s) clearly described?

Yes

Are the datasets clearly presented in a useable and accessible format?

Yes

Are the protocols appropriate and is the work technically sound?

Yes

Reviewer Expertise:

Comparative genomics

I confirm that I have read this submission and believe that I have an appropriate level of expertise to confirm that it is of an acceptable scientific standard.
